# Disruption of Beta-Cell Mitochondrial Networks by the Orphan Nuclear Receptor Nor1/Nr4a3

**DOI:** 10.3390/cells9010168

**Published:** 2020-01-09

**Authors:** Anne-Françoise Close, Nidheesh Dadheech, Hélène Lemieux, Qian Wang, Jean Buteau

**Affiliations:** 1 Department of Agricultural, Food and Nutritional Science, University of Alberta, Edmonton, AB T6G 2E1, Canada; 2 Faculty Saint-Jean, Department of Medicine, University of Alberta, Edmonton, AB T6C 4G9, Canada

**Keywords:** diabetes, Nr4a, mitochondria, mitophagy, nuclear receptor, apoptosis, cytokines

## Abstract

Nor1, the third member of the Nr4a subfamily of nuclear receptor, is garnering increased interest in view of its role in the regulation of glucose homeostasis. Our previous study highlighted a proapoptotic role of Nor1 in pancreatic beta cells and showed that Nor1 expression was increased in islets isolated from type 2 diabetic individuals, suggesting that Nor1 could mediate the deterioration of islet function in type 2 diabetes. However, the mechanism remains incompletely understood. We herein investigated the subcellular localization of Nor1 in INS832/13 cells and dispersed human beta cells. We also examined the consequences of Nor1 overexpression on mitochondrial function and morphology. Our results show that, surprisingly, Nor1 is mostly cytoplasmic in beta cells and undergoes mitochondrial translocation upon activation by proinflammatory cytokines. Mitochondrial localization of Nor1 reduced glucose oxidation, lowered ATP production rates, and inhibited glucose-stimulated insulin secretion. Western blot and microscopy images revealed that Nor1 could provoke mitochondrial fragmentation via mitophagy. Our study unveils a new mode of action for Nor1, which affects beta-cell viability and function by disrupting mitochondrial networks.

## 1. Introduction

Type 2 diabetes is characterized by a progressive deterioration of beta-cell mass and function, leading to a relative deficit in insulin secretion [1,2]. Significant reduction in beta-cell mass has been reported in individuals with type 2 diabetes [3,4], an effect that has been attributed at least in part to increased beta-cell apoptosis [3,5]. However, our understanding of the molecular mechanisms contributing to beta-cell demise in the etiology of diabetes remains incomplete.

Our recent study, investigating the biological functions of Nr4a nuclear receptors in pancreatic beta cells, characterized a key role for the orphan nuclear receptor Nor1 in the regulation of functional beta-cell mass [6]. Indeed, we reported that Nor1-knockout (KO) animals displayed increased beta-cell mass and improved glucose tolerance. In our study, genetic manipulations of Nor1 in beta-cell lines and isolated human islets demonstrated a proapoptotic role of Nor1 in beta cells. Consistently, Nor1 expression was upregulated by cytokines and siRNA-mediated knockdown of Nor1 prevented cytokine-induced beta-cell death. Moreover, Nor1 expression was robustly elevated in islets from type 2 diabetic donors, suggesting that Nor1 could participate in the deterioration of functional beta-cell mass that causes the disease. However, the precise mechanism by which Nor1 provokes beta-cell death remains unexplored. Interestingly, single-nucleotide polymorphisms (SNPs) located within the Nor1 locus have been associated with increased insulin secretion in both nondiabetic and diabetic individuals [7,8], but the biological actions of Nor1 in beta cells remain controversial, with studies describing Nor1 as both a positive [7] and negative [9] regulator of insulin secretion.

Nr4a family members have no known endogenous ligands and are thus considered orphan receptors [10]. Their regulation has been described to be dependent on post-transcriptional modifications and changes in subcellular localization. For instance, in cancer cells, nuclear Nur77 has been shown to promote cell growth [11], whereas its phosphorylation-dependent mitochondrial translocation causes conformational changes in Bcl2 that expose its BH3 death domain, resulting in cytochrome c release and apoptosis [11,12,13,14,15,16]. Nor1 has been documented to act in a similar fashion in thymocytes [17], but to our knowledge, its subcellular localization has not been investigated in beta cells.

Considering the increased interest in Nor1 as a molecular target in type 2 diabetes, we herein sought to investigate its subcellular localization and define the precise mechanism by which it affects beta-cell survival and function.

## 2. Materials and Methods

### 2.1. Reagents

RPMI 1640, fetal bovine serum, L-glutamine, and all other cell culture reagents were purchased from Thermo Fisher Scientific (Burlington, ON, USA). Cytokines were purchased from Millipore Sigma (Oakville, ON, USA).

### 2.2. Cell Culture

INS832/13 cells (passages 57–80, kind gift from Dr. Marc Prentki (Montreal, QC, Canada)) were grown in RPMI 1640 medium containing 11 mM glucose and supplemented with 10 mM HEPES, 10% heat-inactivated fetal bovine serum, 2 mM L-glutamine, 1 mM sodium pyruvate, and 50 µM β-mercaptoethanol at 37 °C in a humidified 5% CO_2_ atmosphere (as originally described in [18]). Our cytokine treatment consisted of a combination of IL-1 (10 ng/mL) and IFNγ (10 ng/mL).

### 2.3. Human Islets

Human islets were purchased from the Alberta Diabetes Institute Islet Core at the University of Alberta with the assistance of the Human Organ Procurement and Exchange Program and the Trillium Gift of Life Network, who provide donor pancreata for research. All experiments were approved by the Human Research Ethics Board at the University of Alberta (protocol no. 00036707). For mitophagy quantification, islets from four different healthy (nondiabetic) donors were used. Donor characteristics are shown in Table 1 below. Islets were cultured in 11 mM glucose RPMI medium supplemented with 10 mM HEPES, 10% heat-inactivated fetal bovine serum, 2 mM L-glutamine, 1 mM sodium pyruvate, 1% penicillin–streptomycin, and 50 µM β-mercaptoethanol at 37 °C in a humidified 5% CO_2_ atmosphere.

### 2.4. Plasmid Transfection

pCMV-Nor1-GFP, pCMV-Nor1-Flag, peGFP-LC3, and pcDNA3.1 were obtained from Origene (Rockville, MD, USA). For INS832/13 cells, plasmids were introduced at a concentration of 5 µg of DNA for 6 × 10^6^ cells via nucleofection (Lonza, Allendale, NJ, USA). Transfection efficiency was >90% with this method. We obtain a 3–4-fold increase in Nor1 protein levels after transfection with Nor1-GFP or Nor1-Flag [6]. For human islets, cells were dispersed using 0.05% trypsin-EDTA and transfected the following day using Lipofectamine 2000 (Thermo Fisher Scientific) according to the manufacturer’s protocol. We obtained ~50% transfection efficiency using Lipofectamine. Cells were assayed 30 h post-transfection.

### 2.5. Mitochondria Staining

Transfected cells were seeded onto 18 mm poly-L-lysine-coated (Millipore Sigma) coverslips (2 × 10^6^ cells per coverslip). Thirty hours post-transfection, they were incubated in the presence or absence of cytokines for 1 h. Media was then changed to RPMI 1640 containing 25 nM Mitotracker^®^ Red CMXRos (Molecular Probes, Eugene, OR, USA) and 10 µg/mL Hoechst 33342 (BD Bioscience, San Jose, CA, USA) with or without 20 µM FCCP (Cayman Chemical, Ann Arbor, MI, USA) for 15 min. The cells were then washed once with PBS. Live cell images were acquired on a WaveFX spinning disk confocal microscope with a Hamamatsu C9100–50 EMCCD camera and analyzed using the Volocity imaging software (version 6.1.2, PerkinElmer, Waltham, MA, USA).

### 2.6. Glucose Oxidation

Glucose oxidation was measured as described before [19] in nonventilated 25 cm^2^ cell culture flasks. Briefly, cells were preincubated in 2 mM glucose KRB medium for 30 min before being treated with 2, 7, or 11 mM glucose. To each flask, 5 µCi of [14C(U)]-D-glucose (Moravek Biochemicals, Brea, CA, USA) was added. All metabolic reactions were stopped after 45 min by adding H_2_SO_4_ to the media. 14C-CO_2_ was captured using fiberglass fiber filters and counted using a scintillation counter.

### 2.7. Glucose Uptake

Glucose uptake was assessed using a commercial kit (Glucose Uptake-Glo™Assay, Promega, Madison, WI, USA). Cells were treated as described above in Section 2.5 for glucose oxidation assays, except that 5% of the total glucose of each condition was 2-deoxyglucose.

### 2.8. ATP Production

Cells were washed with cold PBS and lysed in a buffer containing 10 mM Tris pH 7.5, 100 mM NaCl, 1 mM EDTA, and 0.01% Triton X100. ATP was quantified using the ATP Determination Kit (Molecular Probes).

### 2.9. Mitochondrial Membrane Potential

Cells were treated in the presence or the absence of cytokines for 4 h before the assay was performed. FCCP (20 µM) was used as a negative control and 25 mM glucose as a positive control. Mitochondrial membrane potential was then evaluated using the JC-1 Mitochondrial Membrane Potential Assay Kit (Cayman chemicals, Ann Arbor, MI, USA).

### 2.10. Mitochondrial Ultrastructure

Transfected cells were prefixed in 4% glutaraldehyde in 0.2 M sodium cacodylate buffer, postfixed in 1% osmium tetroxide (OsO4) in 0.1 M sodium cacodylate buffer, dehydrated in an ethyl alcohol series, embedded with epoxy resin, and thermally polymerized as previously described [20,21]. Ultrathin sections (70 nm) were cut by an ultramicrotome (Leica Microsystems, Richmond Hill, ON, Canada) and then stained with 4% uranyl acetate and Reynold’s lead citrate. The contrasted sections were imaged using a Hitachi H-7650 transmission electron microscope at 80 kV equipped with a 16-megapixel EMCCD camera (XR111, Advanced Microscopy Technique, Woburn, MA, USA).

### 2.11. Quantification of Mitophagy

Fluorescence microscopy: Cells were transfected with peGFP-LC3 and with either Nor1-Flag or a control empty vector 30 h after transfection; cells were imaged as described in Section 2.4. The colocalization of peGFP-LC3 positive vacuoles and mitochondria was determined using the Imaris software (version 8.4, Bitplane, Concord, MA, USA). Electron microscopy: The number of autophagic vacuoles targeting the mitochondria was determined as described before [22]. Briefly, we examined three grid squares per condition. In each square, images were taken at 400× to assess whole cell areas and at 12,000× to count the number of autophagic vacuoles.

### 2.12. Western Blot

Cells were lysed in a buffer containing 50 mM Tris (pH 8.0), 150 mM NaCl, and 1% *v*/*v* Triton X-100 and supplemented with protease inhibitors (Complete Mini, Roche Diagnostics, Mannheim, Germany). Cytosolic and mitochondrial protein extracts were obtained using a cell fractionation kit (Abcam, Toronto, ON, Canada). Protein concentrations were determined by BCA protein assay. Equal amounts of heat-denatured proteins from each treatment group were run on Novex 10% Tris-glycine gels (Thermo Fisher Scientific) and electrically transferred to nitrocellulose membranes. After blocking for 1 h at room temperature with 1% BSA, membranes were incubated overnight at 4 °C with primary antibodies. The next day, membranes were incubated with horseradish-peroxidase-linked secondary antibodies followed by exposure to Amersham ECL Western Blotting Detection Reagents (GE Healthcare, Mississauga, ON, Canada) and film development. The primary antibodies used in our studies were rabbit anti-VDAC antibody (Cell Signaling, Danvers, MA, USA), mouse monoclonal anti-tubulin (Santa Cruz Biotechnology, Dallas, TX, USA), and rabbit anti-LC3 (Novus Biologicals, Oakville, ON, Canada).

### 2.13. Citrate Synthase Activity

Cells were harvested in a solution containing methylsulfonylmethane (MSM)/EDTA supplemented with 1% sodium cholate hydrate. Citrate synthase activity was then evaluated by measuring the conversion of 5,5′-dithiobis-(2-nitrobenzoic acid) (DTNB, 0.1 mM) into 2-nitro-5-benzoic acid (TNB), which absorbs specifically at 412 nm. The reaction was carried out in a buffer containing 0.25% Triton X100, 0.5 mM oxaloacetate, and 0.31 mM acetyl-CoA. Results were normalized to total protein content of the cells.

### 2.14. High-Resolution Respirometry

Cellular aerobic respiration was measured using high-resolution respirometry (Oxygraph-2k, Oroboros Instruments, Innsbruck, Austria) [23], as we have performed before [24]. In brief, the oxygraph was calibrated at 37 °C per the manufacturer’s instructions with each chamber filled with 2 mL of mitochondrial respiration medium 05 (MIR05) containing 110 mM sucrose, 60 mM K-lactobionate, 0.5 mM EGTA, 0.1% BSA, 3 mM MgCl_2_, 20 mM taurine, 10 mM KH_2_PO_4_, and 20 mM HEPES [25], which was magnetically stirred at 500 rpm. DatLab 4 software (Oroboros Instruments, Innsbruck, Austria) was used for data acquisition and analysis. Equal numbers of transfected cells (1 million cells per condition) were rinsed twice with MIR05 and transferred in each oxygraph chamber. After measurement of routine respiration in MIR05 and permeabilization of the cell membranes with digitonin [26], the following substrates and inhibitors were added (final concentration in the chamber): glutamate (10 mM), malate (5 mM), and pyruvate (5 mM) as Complex I (CI)-linked substrates; succinate (10 mM) as Complex II (CII)-linked substrates; rotenone (0.5 μM) and antimycin A (2.5 μM) as CI and CIII inhibitors; ascorbate (0.5 mM) and tetramethylphenylenediamine (TMPD, 2 mM) as CIV-linked substrates. Mitochondrial respiration was corrected for oxygen flux due to instrumental background and for residual oxygen consumption after inhibition of Complexes I and III with rotenone and antimycin A, respectively.

### 2.15. Statistical Analysis

Data are presented as mean ± SEM. Statistical analyses were performed with GraphPad Prism^®^ (GraphPad Software, San Diego, CA, USA) using ANOVA followed by Bonferroni’s post hoc test. *p*-values < 0.05 were considered statistically significant.

## 3. Results

### 3.1. Nor1 Translocates to the Mitochondria in INS832/13 Cells Exposed to Proinflammatory Cytokines

**Figure 1:**
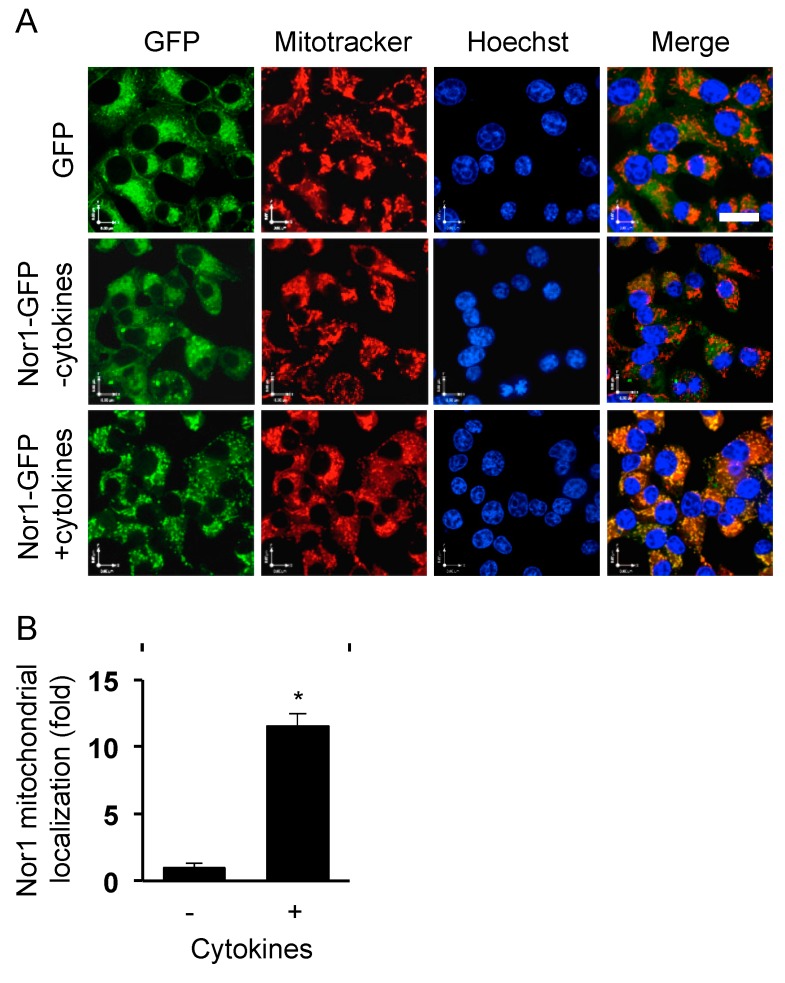
Nor1 translocates to the mitochondria in response to cytokines in INS832/13 cells. (**A**) Images show representative INS832/13 cells transfected with either control GFP or Nor1-GFP and subsequently incubated in the presence or in the absence of cytokines (10 ng/mL IL-1β and 10 ng/mL IFNγ) for 1 h. Mitochondria were stained in red (Mitotracker) and nuclei in blue (Hoechst). (**B**) Graph shows the quantification of Nor1-GFP localization at the mitochondria as determined by the overlap of the green and red signals. Scale bar = 20 μm. Results are mean ± SEM of three independent experiments, * *p* < 0.05 versus control vector.

Our previous study characterized the nuclear receptor Nor1 as a novel mediator of cytokine-induced beta-cell apoptosis mass [6]. To gain insights into the mechanism, we first sought to investigate the subcellular localization of Nor1 in beta cells. Because of the dearth of reliable Nor1 antibodies, we overexpressed a Nor1-GFP protein in INS832/13 cells cultured in the absence or presence of a cocktail of cytokines comprising IL-1 (10 ng/mL) and IFNγ (10 ng/mL). Mitochondria and nuclei were stained with Mitotracker Red and DAPI, respectively. In untreated (control) cells, Nor1-GFP showed a diffuse cytosolic signal, which did not colocalize with the nucleus or the mitochondria (Figure 1). However, upon cytokine treatment, Nor1-GFP was found to rapidly translocate and accumulate at the mitochondria, as detected by the overlap of the green and red signals. Surprisingly, we could not detect Nor1-GFP in the nucleus in any of the time points tested (up to 24 h). Nuclear exclusion of Nor1 was unexpected since it raised questions about its presumed role as a transcription factor in beta cells. Be that as it may, the translocation of Nor1 at the mitochondria in response to cytokines prompted us to study its effects on mitochondrial function.

### 3.2. Nor1 Affects Mitochondrial Function and Reduces Insulin Secretion in INS832/13 Cells

**Figure 2:**
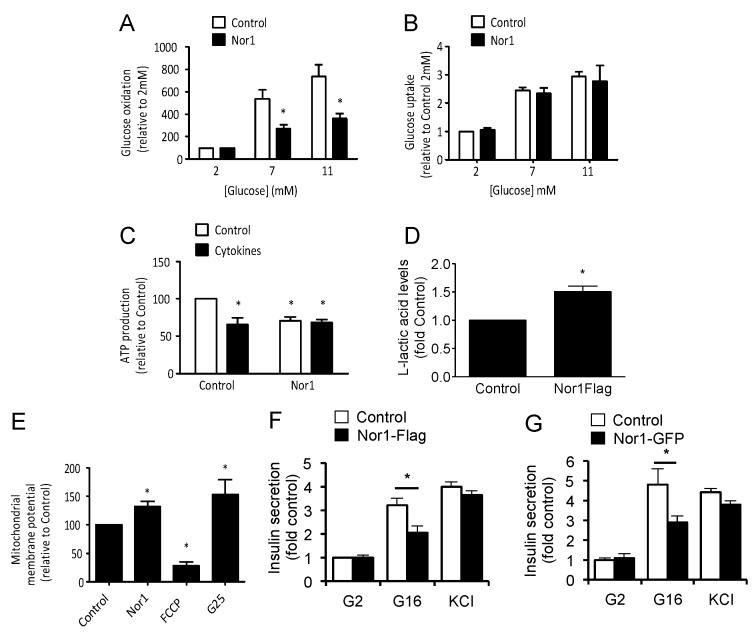
Nor1 impairs glucose oxidation and insulin secretion in beta cells. Glucose oxidation (**A**) and glucose uptake (**B**) were measured in INS832/13 cells transfected with Nor1-Flag (black bars) or a control flag vector (white bars) and exposed to 2, 7, or 11 mM glucose for 45 min. (**C**) ATP levels measured in INS832/13 cells transfected with Nor1-Flag or a control flag vector and cultured at 11 mM glucose in the presence (black bars) or absence (white bars) of cytokines. (**D**) Lactic acid levels were measured in cells transfected with Nor1-Flag or a control flag vector and cultured at 11 mM glucose. (**E**) Mitochondrial membrane potential was determined by evaluating JC-1 fluorescence in INS832/13 cells transfected with Nor1-Flag or a control flag vector. Cells were exposed to 20 µM FCCP for 15 min and 25 mM glucose for 4 h (G25) as negative and positive controls, respectively. (**F**) Insulin secretion was measured in cells transfected with Nor1-Flag (black bars) or a control flag vector (white bars) at low (2 mM glucose, G2) or stimulatory glucose concentrations (16 mM glucose, G16) by ELISA. KCl (35 mM) was used to maximally depolarize the cells. Results are expressed as fold control (control vector at 2 mM glucose). (**G**) Insulin secretion was measured in cells transfected with Nor1-GFP compared to GFP control as described in (**F**) above. All results are represented as mean ± SEM of three separate experiments. * *p* ˂ 0.05 versus control.

In beta cells, mitochondrial function plays a critical role in the regulation of insulin secretion. In particular, glucose-stimulated oxidative ATP production causes a rise in the cytosolic ATP/ADP ratio, which triggers a series of electrophysiological events that provoke insulin exocytosis. We thus investigated the potential effect of Nor1 on glucose metabolism, ATP production, mitochondrial membrane potential, and insulin secretion. Nor1 significantly blunted glucose oxidation in cells exposed to intermediate (7 mM) or high (11 mM) glucose concentrations (Figure 2A). This effect was not due to a reduction in glucose uptake, which remained unaffected by Nor1 overexpression (Figure 2B). Consistently, with its action on glucose oxidation, Nor1 decreased glucose-stimulated ATP production by ~30%, an effect that was not additive to the effect of cytokines (Figure 2C). The decrease in ATP production was concomitant with an increase in lactic acid levels (Figure 2D), suggesting that pyruvate conversion to lactic acid replaces pyruvate oxidation in the mitochondria.

Surprisingly, Nor1 significantly increased mitochondrial membrane potential by ~30% (Figure 2E). This is reminiscent of a model [27,28,29] in which hyperpolarization of the inner mitochondrial membrane in proapoptotic conditions is proposed to provoke mitochondrial matrix swelling and ruptures in the outer membrane. Nor1-induced changes in glucose oxidation and ATP production translated into inhibition of glucose-stimulated insulin secretion (Figure 2F,G). Nor1 did not affect insulin secretion in response to KCl-induced depolarization, indicating that the inhibitory effects of Nor1 on insulin secretion are due to impairment of glucose oxidation and/or mitochondrial function and not through an action on the exocytotic machinery.

**Figure 3:**
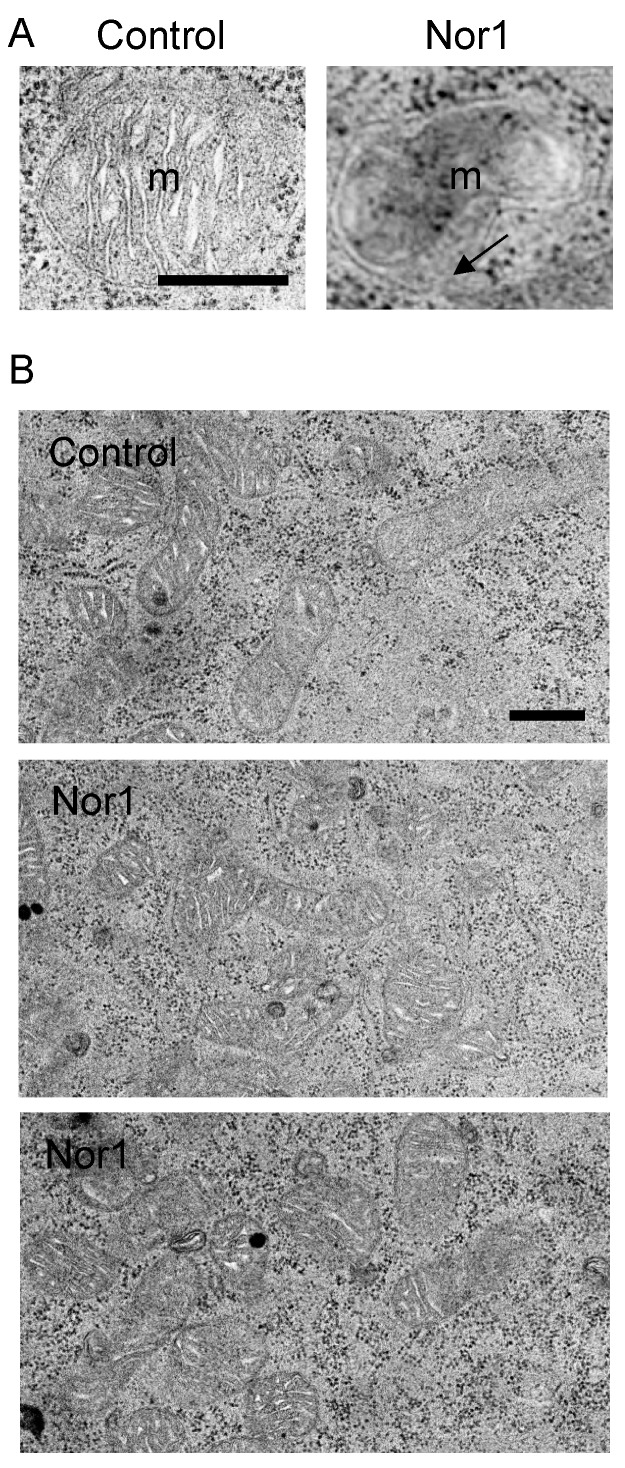
Representative TEM images of mitochondria from INS832/13 cells transfected with Nor1 versus control. (**A**) Nor1 transfected cells displayed occasional hernia and breaks in the outer mitochondrial membrane (indicated by the arrow). m, mitochondria. (**B**) Representative lower magnification images of control and Nor1-overexpressing cells. Scale bars = 500 nm.

We next examined mitochondria morphology by TEM in INS832/13 cells transfected with Nor1 or its control vector (Figure 3). Nor1-expressing cells displayed mitochondrial matrix swelling and subsequent herniation through breaks in the outer membrane. These features were absent in control cells. It is known that outer mitochondrial membrane ruptures allow for the release of cytochrome c from the intermembrane space into the cytosol. Yet, we have recently shown that Nor1 does significantly increase cytochrome c release to cause apoptosis in beta cells [6].

### 3.3. Nor1 Induces Mitochondrial Fractionation in Beta Cells

**Figure 4:**
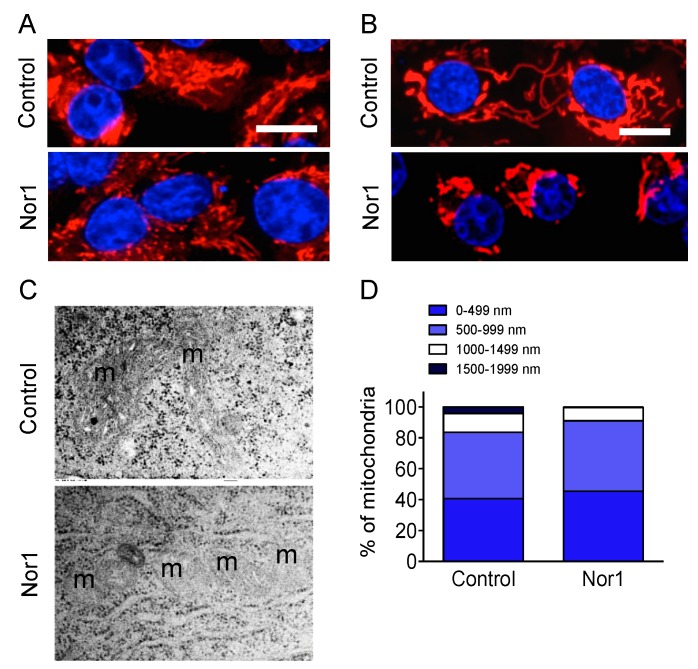
Nor1 induces mitochondrial fractionation in beta cells. Representative images of INS832/13 cells (**A**) or dispersed human islet cells (**B**) transfected with Nor1-Flag (bottom) or a control flag vector (top). Mitochondria were stained using Mitotracker Red, and the nuclei using Hoechst. Scale bars = 10 μm. (**C**) TEM images showing mitochondrial fractionation in INS cells transfected with Nor1 (bottom) compared to a control vector (top). m denotes mitochondria. (**D**) Quantification of the number of mitochondria by size groups in TEM images.

We then performed morphological studies to confirm a potential disrupting action of Nor1 on the mitochondrial networks (Figure 4). Our fluorescence microscopy images showed a strong disruption of the networks in Nor1-expressing cells. Indeed, Nor1-transfected INS832/13 cells (Figure 4A) and human islet cells (Figure 4B) displayed decreased mitochondrial networked areas and increased punctate staining. Moreover, mitochondrial fractionation was also observed in TEM images (Figure 4C). Measurements of mitochondrial lengths in fluorescence microscopy images revealed that Nor1 induces a shift in the distribution of mitochondria towards smaller sizes (Figure 4D). Indeed, there was an accumulation of smaller mitochondria (<499 nm in length) in cells transfected with Nor1 compared with control. On the contrary, large mitochondria of >1500 nm were virtually absent in Nor1-expressing cells. We next evaluated if the mitochondria were subjected to mitophagy.

### 3.4. Nor1 Increases Mitophagy in Beta Cells

**Figure 5:**
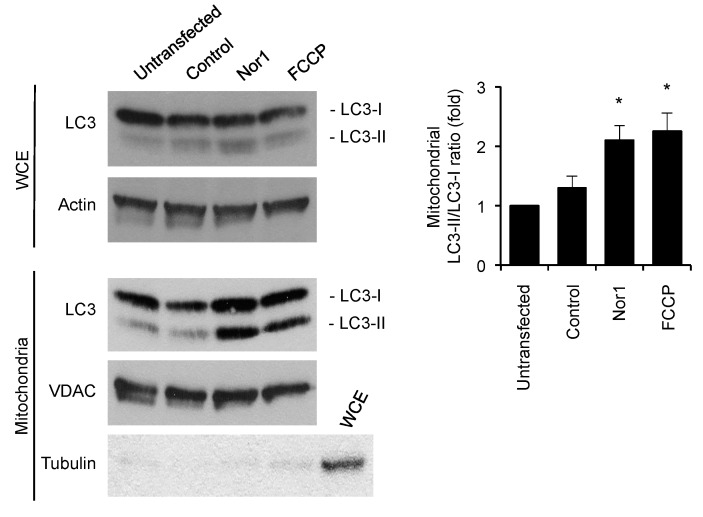
Nor1 increases the conversion of LC3 in beta cells. Representative Western blot images of LC3-I and -II in whole-cell extracts (WCEs) or mitochondrial fractions of untransfected INS832/13 cells, or cells transfected with Nor1-Flag and a control flag vector. FCCP was used as positive control. Actin, tubulin, and VDAC were used to demonstrate equal loading and assess the purity of mitochondrial fractions. The graph shows densitometry analysis of the mitochondrial LC3-I/LC3-II ratios. Results are mean ± SEM of three independent experiments, * *p* < 0.05 versus control vector.

**Figure 6:**
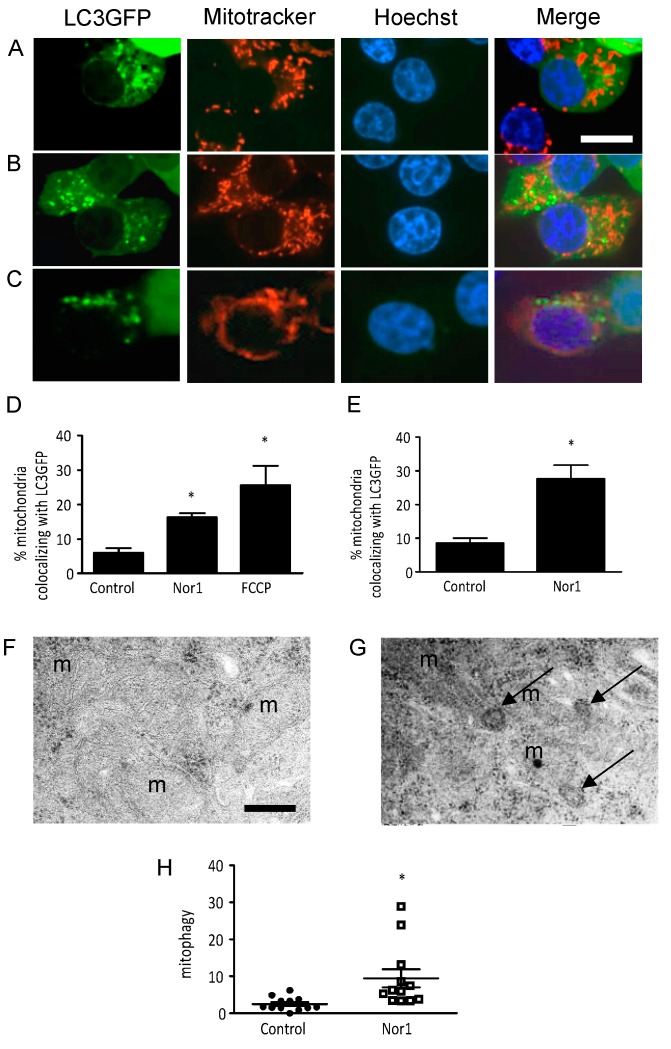
Nor1 increases mitophagy in beta cells. (**A**–**C**) Immunofluorescence images of INS832/13 cells transfected with LC3-GFP along with Nor1 (**B**) or a control vector (**A**,**C**). FCCP (20 µM, 15 min) was used as a positive control in (**C**). Scale bar = 10 μm. (**D**) Quantification of the colocalization between LC3-GFP and mitochondria in INS832/13 cells (*n* = 3). Results are expressed as the percentage of mitochondria (red signal) colocalizing with LC3-GFP in each group. (**E**) Quantification of the colocalization between LC3-GFP and mitochondria in dispersed human islets cells (*n* = 4). Results are expressed as fold change over untreated control cells. (**F**,**G**) TEM images of mitochondria in control INS832/13 cells (**F**) and cells transfected with Nor1 (**G**). Arrows indicate autophagosomes. Scale bar = 250 nm. (**H**) Quantification of the number of autophagosomes targeting mitochondria per cell area in TEM images. All results are represented as mean ± SEM; *, *p* ˂ 0.05 versus control.

We first assessed mitophagy by Western blot. We investigated LC3 conversion in whole-cell extracts and mitochondrial fractions of INS832/13 cells transfected with Nor1 or a control vector (Figure 5). FCCP was used as a positive control. Whereas Nor1 did not significantly affect LC3 levels in whole-cell extracts, it induced an accumulation of LC3-II in mitochondrial fractions, providing evidence that Nor1 could induce mitophagy in beta cells. The effect of Nor1 was similar to that of FCCP. VDAC and tubulin were used to assess the quality of cell fractionation. We detected equal amounts of VDAC in all mitochondrial samples, while tubulin was absent. To further document Nor1 action on mitophagy, we quantified the colocalization of mitochondria (in red) with a LC3-GFP construct (green) in INS832/13 cells transfected with Nor1 or a control vector (Figure 6A,B), respectively, or exposed to FCCP (Figure 6C). We detected a significant 2-fold increase in the colocalization of the green and red signals in Nor1-expressing INS832/13 cells (Figure 6D). Similar experiments in dispersed human islet cells revealed a 2–3-fold increase in mitochondrial LC3-GFP localization (Figure 6E). We also quantified the number of autophagosomes targeting the mitochondria in our TEM images (Figure 6F,G) and detected a significant increase (~4-fold) in cells transfected with Nor1 (Figure 6H).

### 3.5. Nor1 and Mitochondrial Content in Beta Cells

**Figure 7:**
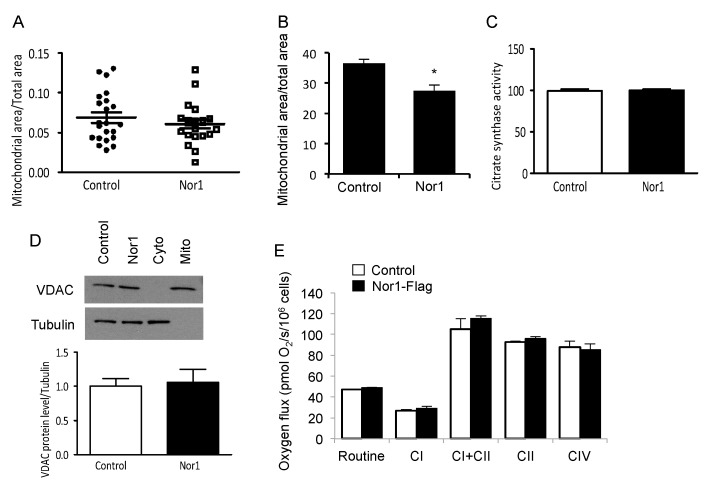
Nor1 and mitochondrial content in beta cells. (**A**) Relative mitochondrial area was determined from TEM images of INS832/13 cells transfected with Nor1-Flag or a control flag vector and normalized to total cell area. (**B**) Relative mitochondrial area was determined from fluorescence images of INS832/13 cells transfected with Nor1-GFP or a control GFP vector using Mitrotracker Red and normalized to total cell area. (**C**) Citrate synthase activity was measured in control cells (white bar) or cells overexpressing Nor1 (black bar) (*n* = 4). (**D**) VDAC protein levels were measured by Western blot in INS cells transfected with Nor1-Flag or a control flag vector. Cytoplasmic (Cyto) and mitochondrial (Mito) fractions were used as negative and positive controls, respectively. Results were normalized to tubulin (*n* = 5). (**E**) Sequential respiratory states in INS832/13 cells transfected with Nor1-Flag or a control vector (*n* = 4). The protocol includes the following steps: Routine (basal respiratory state), Complex-I (CI; oxygen flux after addition of malate, pyruvate, and glutamate), CI + CII (after addition of succinate), CII (after inhibition of CI by rotenone), and CIV (after addition of TMPD and ascorbate). Respiration is expressed per million cells. All results are represented as mean ± SEM; *, *p* ˂ 0.05 versus control.

Next, we sought to confirm that the increase in mitophagy induced by Nor1 translated into decreased mitochondrial content. Unfortunately, we obtained conflicting results depending on the methods used. For instance, the mitochondrial area relative to total cell area was not significantly altered in cells overexpressing Nor1 in our TEM images (Figure 7A). However, it was significantly decreased in our immunofluorescence images (Figure 7B). Additionally, Nor1 did not change citrate synthase activity (Figure 7C) or VDAC protein levels compared to controls (Figure 7D). We initially expected that Nor1 would decrease mitochondrial content due to its effect on mitophagy, so these results were unexpected. It is possible that Nor1 increases the mitochondrial turnover rate in beta cells to keep the mitochondrial content unchanged despite its effect on mitophagy. Another plausible scenario is that mitophagy precedes the changes in mitochondrial content and that we were too early to detect significant changes in content.

To test whether Nor1 affects energy metabolism by acting on specific respiratory complexes, we performed high-resolution respiratory analyses in permeabilized cells transduced by Nor1 or a control vector in the presence of inhibitors or substrates feeding electrons into specific mitochondrial respiratory complexes (Figure 7E). Routine respiratory rates were identical between control cells and Nor1-overexpressing cells. Also, we could not detect any significant differences between the respiratory capacity of individual complexes in cells with or without Nor1 gain-of-function. Since Nor1 was found to reduce glucose oxidation and ATP production in beta cells (Figure 2A−C), these results suggest that Nor1 could act predominantly by diverting pyruvate to lactate production, as shown in Figure 2D.

## 4. Discussion

Nr4as are garnering considerable interest for their potential implication in metabolic diseases, in particular diabetes [10,30]. However, their biological actions in pancreatic beta cells remain incompletely understood. Our recent work characterized Nor1 as a mediator of cytokine-induced beta-cell death [6]. Indeed, Nor1 gain-of-function provoked beta-cell apoptosis whereas Nor1 loss-of-function prevented cytokine-induced apoptosis. Moreover, Nor1-KO animals displayed higher beta-cell mass and improved glucose tolerance. Finally, we also showed that Nor1 expression was increased in type 2 diabetic islets, suggesting that Nor1 could play a role in the etiology of diabetes.

We herein documented the subcellular localization of Nor1 in untreated and cytokine-treated cells. Surprisingly, we discovered that in beta cells, activated Nor1 undergoes mitochondrial translocation. In our conditions, we could not detect nuclear Nor1, suggesting that the “nuclear receptor” may not act primarily as a transcription factor in beta cells. This would confer a distinct biological role for Nor1 as compared with the other members of the Nr4a family. Indeed, Nur77 and Nurr1 have been shown previously to translocate to the nucleus and regulate transcription in response to various stressors in beta cells [31,32].

A similar honing of Nor1 to the mitochondria has been described in immune cells [11,12,13,14,15,16,17]. In this model, Nor1 was found to interact with Bcl2 to expose its BH3 domain and trigger the intrinsic pathway of apoptosis. However, the biological outcome appears to be different in beta cells because the canonical pathway of intrinsic apoptosis involves the dissipation of the mitochondrial membrane potential [33]. Our data indicate that Nor1 rather increases mitochondrial membrane potential. An alternative model exists and proposes that proapoptotic stimuli could induce mitochondrial hyperpolarization, resulting in an osmotic imbalance capable of provoking outer mitochondrial membrane rupture and the release of intermembrane space proteins such as cytochrome c to induce cell death [27,28,29]. Our results dovetail with this alternative model and suggest that Nor1 causes beta-cell death via both impairment of beta-cell function and disruption of the mitochondrial network. Of note, mitochondrial membrane potential was measured using the JC-1 ratiometric dye, the fluorescence of which may change in response to factors other than mitochondrial membrane potential [34]. Thus, results obtained with JC-1 must be interpreted with caution.

Our results suggest that Nor1 could affect beta-cell energy metabolism and cause changes to mitochondrial function. Altogether, these changes translate into blunted glucose oxidation, reduced ATP production, and, consequently, inhibition of insulin secretion. Interestingly, Nor1 thus reiterates the effects of cytokines, which were recently shown to suppress pyruvate oxidation and inhibit insulin secretion in beta cells [35]. In our model, Nor1-induced impairment of mitochondrial function and energy production was concomitant with the induction of morphological changes at the mitochondria, including increased mitochondrial fragmentation and the induction of mitophagy. These observations are relevant, since mitochondrial dynamics govern the maintenance of optimal mitochondrial function [36] and it has been previously demonstrated that disruption of the mitochondrial network leads to reduced glucose oxidation and ATP production [37]. Moreover, mitochondrial fragmentation also actively participates in the triggering of apoptosis [38]. Thus, our results linking Nor1 to mitochondrial fragmentation provide a molecular mechanism by which Nor1 could both stimulate beta-cell apoptosis and impair beta-cell function, two defects that participate in the development of diabetes.

Mitochondrial dynamics also dictate the removal of these organelles by autophagy [39]. Small, rounded, fragmented mitochondria are indeed targeted by autophagosomes. We thus quantified mitophagy and detected an accumulation of double-membrane vacuoles targeting the mitochondria in Nor1-expressing cells. Interestingly, the number of autophagosomes has been shown to be increased in the beta cells of diabetic individuals compared with nondiabetic subjects [40]. To our knowledge, the role of Nr4a members in autophagy remains unexplored. A single publication reported that Nur77 could induce autophagic cell death following its translocation to the mitochondria [15,41].

## 5. Conclusions

In conclusion, our study demonstrates for the first time that the nuclear receptor Nor1 translocates to the mitochondria in beta cells to disrupt mitochondrial networks, a process known to regulate both beta-cell function and viability. These results are important considering that we previously detected an upregulation of Nor1 in type 2 diabetic islets. We thus believe that Nor1 constitutes a potential molecular target to improve or maintain functional beta-cell mass.

## Figures and Tables

**Table 1:** Pancreatic islet donor characteristics.

	Sex	Age	BMI
1	Female	50	44
2	Female	68	21.7
3	Male	62	23.8
4	Male	61	29.6
